# The digital transformation of nursing practice: an analysis of advanced IoT technologies and smart nursing systems

**DOI:** 10.3389/fmed.2024.1471527

**Published:** 2024-11-29

**Authors:** Boyuan Wang, Xiali Shi, Xihao Han, Gexin Xiao

**Affiliations:** ^1^Beijing Xiaotangshan Hospital, Beijing, China; ^2^University of Shanghai for Science and Technology, Shanghai, China; ^3^National Institute of Hospital Administration, Beijing, China

**Keywords:** Internet of Things, healthcare, smart platforms, technical challenges, international cooperation, policy recommendations

## Abstract

Facing unprecedented challenges due to global population aging and the prevalence of chronic diseases, the healthcare sector is increasingly relying on innovative solutions. Internet of Things (IoT) technology, by integrating sensing, network communication, data processing, and security technologies, offers promising approaches to address issues such as nursing personnel shortages and rising healthcare costs. This paper reviews the current state of IoT applications in healthcare, including key technologies, frameworks for smart nursing platforms, and case studies. Findings indicate that IoT significantly enhances the efficiency and quality of care, particularly in real-time health monitoring, disease management, and remote patient supervision. However, challenges related to data quality, user acceptance, and economic viability also arise. Future trends in IoT development will likely focus on increased intelligence, precision, and personalization, while international cooperation and policy support are critical for the global adoption of IoT in healthcare. This review provides valuable insights for policymakers, researchers, and practitioners in healthcare and suggests directions for future research and technological advancements.

## 1 Introduction

Medical care involves the utilization of pertinent clinical data in conjunction with physicians to deliver effective treatments (1). Globally, healthcare systems face challenges such as an aging population, the prevalence of chronic diseases, shortages of healthcare personnel, rising medical costs, and increasing patient expectations. According to United Nations data, population aging is a distinct global trend of our times, In 2021, there were 761 million people aged 65 and over globally, and this figure is projected to rise to 1.6 billion by 2050. The population aged 80 and above is growing even more rapidly. Regarding cardiovascular diseases, in 2016, 8.4 million people died from cardiovascular diseases in South Africa (one of the BRICS nations). Between 1992 and 2016, the age-standardized mortality rate for cardiovascular diseases in the BRICS countries declined by −17%, which was less than the decline seen in North America (−39%) (2). For chronic respiratory diseases, between 1990 and 2017, the incidence of CRD, COPD, pneumoconiosis, and asthma declined, whereas the incidence of interstitial lung disease and sarcoidosis showed an upward trend. Elderly individuals are particularly susceptible to CRD, except for asthma. The acceleration of population aging and increased exposure to risk factors such as particulate matter pollution will contribute to the growing burden of chronic respiratory diseases globally (3). The International Labour Organization (ILO) estimates that by 2030, there will be a global shortage of 18 million healthcare workers (4). To address these issues, the United States enacted legislation in 2009 to promote the comprehensive development of healthcare informatics (5), and China’s 14th Five-Year Plan emphasizes the role of information technology in driving high-quality healthcare (6). The COVID-19 pandemic has further highlighted the importance of quality healthcare (7), while the rapid development of 5G technology has increased the demand for high-quality care, accelerating the progress toward smart healthcare.

The Internet of Things (IoT) offers the potential for intelligent care through the collection of information via sensors and the transmission of this data for the intelligent monitoring and tracking of patients. IoT’s terminal sensing layer, including smart bracelets, remote monitoring devices, and wearable sensors, enables healthcare professionals to obtain real-time health data from patients.

This data includes vital signs such as heart rate, blood pressure, glucose levels, and sleep patterns, which can help in early detection and timely intervention for health issues. Umer et al. (8) utilized IoT to construct an ET-CNN model for remote monitoring of heart rate in cardiac patients with an accuracy of 0.9524. Alshammari (9) employed an IoT-based MQTT model for accurate detection of patients’ vital signs and secure data transmission. Moreover, IoT technologies can be combined with artificial intelligence (AI) and big data analytics to provide personalized health recommendations and treatment plans tailored to each patient’s unique health needs. Astell et al. (10) provided personalized tablets for long-term care residents to enhance their quality of life. Ni Ki et al. (11) used a Latent Dirichlet Allocation model based on IoT to tailor treatment plans for diabetic patients, improving their outcomes. This approach not only enhances the precision and effectiveness of treatments but also empowers patients to better manage their own health, improving their overall quality of life.

The application of Internet of Things (IoT) technologies plays a crucial role in enhancing nursing efficiency and reducing costs. Traditional nursing models often require substantial human resources and time, particularly in geriatric care and chronic disease management. Through automation and intelligent means, IoT technologies can significantly alleviate the burden on healthcare providers and decrease medical expenses (12, 13). Partners HealthCare (14) provided home monitoring for over 3,000 patients with congestive heart failure, resulting in a 44% reduction in hospital readmissions over six years and saving more than $10 million. Mullins et al. (15) found that through the application of IoT in healthcare, consultation times were reduced by over two minutes, and patient out-of-pocket costs decreased by $1,229 to $2,700. Smart pillboxes can remind patients to take their medications on time, ensuring continuity and effectiveness of treatment and reducing complications from missed or incorrect doses (16). Furthermore, El-Saleh et al. (17) noted that the global digital health market was valued at approximately $175 billion in 2019. These smart devices not only improve the efficiency of care but also reduce costs.

The integration of IoT technologies into healthcare has significantly enhanced the quality and safety of care, while also driving the digitization of nursing processes (18). In terms of process digitization, IoT devices have enabled many automated management functions (19, 20). This paper will analyze key IoT technologies, discuss the design framework for a smart IoT nursing platform, and explore future technological advancements.

## 2 Key IoT technologies

IoT (Internet of Things) technologies encompass a range of essential components, including sensing technologies, networking technologies, data processing technologies, and security technologies. Sensing technologies utilize various sensors (such as temperature, humidity, and light sensors) to collect environmental data, forming the foundation of IoT systems. Networking technologies transmit these data via wireless communications (such as Wi-Fi, 5G, and LoRa) and wired communications (such as Ethernet) to cloud or local servers, enabling device-to-device connectivity. Data processing technologies leverage big data analytics and artificial intelligence (AI) to extract valuable information from vast datasets, enhancing system intelligence and automation. Lastly, security technologies ensure data privacy and security, preventing cyberattacks and data breaches, which is a critical aspect of IoT applications. These technologies collectively form the technical framework of IoT, driving its widespread adoption in smart homes, smart cities, and smart healthcare.

### 2.1 Sensor networks and data acquisition

Sensor networks and data acquisition are core technologies in IoT. A sensor network consists of multiple sensor nodes connected through wireless communication protocols (such as Zigbee and LoRa) to enable real-time collection and transmission of environmental data (such as temperature, humidity, and pressure). These data are uploaded to the cloud or local servers, processed through big data analytics and machine learning, and transformed into valuable information for smart decision-making and automated control, improving system efficiency and reliability.

#### 2.1.1 Biomedical sensors and wearable devices

Recent advancements in biomedical sensors and wearable devices focus on addressing challenges such as power supply, sensor sensitivity, selectivity, and communication. Li et al. (21) leveraged the flexibility of liquid metal gallium to seamlessly integrate it with human tissue, enabling real-time health monitoring and early issue detection. Wang et al. (22) reviewed nanomaterials-based stretchable self-powered technologies, achieving intelligent, miniaturized, and wireless wearables. Tricase et al. (23) utilized printed electrode technology to prepare graphite electrodes for monitoring L-lactate and D-glucose, with sensitivities of 1.32 mA/mM and 28.4 μA/mM, respectively. Nguyen et al. (24) developed freeze-dried, cell-free genetic components integrated into flexible materials for biosensors that achieve high sensitivity and specificity without power or liquid handling, suitable for wearable applications.

#### 2.1.2 Environmental sensing sensors in hospital environment monitoring

Environmental sensing sensors play a critical role in hospital environment monitoring, especially in intensive care units and refrigeration facilities. These sensors monitor parameters such as temperature, humidity, carbon dioxide levels, and acoustic noise, providing real-time data to maintain optimal conditions. Yasin et al. (25) conducted real-time monitoring of indoor air quality using IoT sensors, demonstrating adaptability across various work environments. Sivakumar et al. (26) used Ubidots IoT to monitor temperature, humidity, and refrigeration equipment in specialized hospital areas, issuing alerts when limits are exceeded. Abubeker and Baskar (27) employed a wireless sensor network and wireless body area network-assisted biosensors to track volatile organic compounds and other atmospheric constituents, limiting the spread of non-ventilator-associated pneumonia in hospitals, achieving good results in computing, communication, storage, and energy utilization. Wilson et al. (28) used CO_2_ detection to quantify ventilation in hospital environments, effectively reducing the risk of airborne pathogen transmission between healthcare workers and patients. Alabere et al. (29) used the HC-SRO4 ultrasonic sensor to monitor intravenous fluid bags in hospitals, transmitting fluid level information via web, alleviating real-time monitoring issues for intravenous infusions. Liu et al. (30) utilized superconducting material sensors and deep learning for environmental monitoring, detecting humidity levels with a sensing accuracy exceeding 93%.

Although these sensors offer significant benefits, such as real-time monitoring and automatic adjustment, they also possess certain limitations. For instance, wireless sensor networks may encounter issues such as signal interference or battery life constraints. Moreover, some sensors require a continuous data connection, which can present a challenge in remote areas.

#### 2.1.3 Mobile health solutions in remote patient monitoring

Mobile health solutions have made significant strides in remote patient monitoring. Alasmary (31) integrated IoT using the Scalable Digital Health framework for real-time tracking of health parameters, enabling remote patient monitoring through delay-aware edge computing, enhancing accessibility and responsiveness. Raparla et al. (32) used Dozee.ai and Qure.ai devices for remote monitoring of COVID-positive patients, significantly assisting doctors in tracking patients’ physical parameters and reducing healthcare costs. Ren et al. (33) observed response pattern trends in remote patient monitoring for concussed adolescents, indicating that demographic factors, injury history, and time post-injury influence higher evening incidence rates. Kedwan (34) discussed the implementation of a remote physiological monitoring system in cardiac care centers, showcasing the impact of health informatics on improving patient care efficiency. Vaghasiya et al. (35) proposed an integrated FePS3/rGO and Ti3C2 remote healthcare platform for remote health assessment, demonstrating a wearable device for real-time monitoring of physiological parameters, facilitating remote patient monitoring. Overall, these case studies demonstrate the significant impact of mobile health solutions in enhancing remote patient monitoring across various medical domains.

Despite the enhancement of telemonitoring in various healthcare domains through mobile health solutions, there are also several limitations associated with these technologies. For example, the reliability and accuracy of the devices may be affected by environmental factors, and data privacy and security remain critical considerations. Additionally, given the rapid pace of technological advancements, ensuring that the devices are always up-to-date poses another challenge.

### 2.2 Wireless communication technologies

**FIGURE 1:**
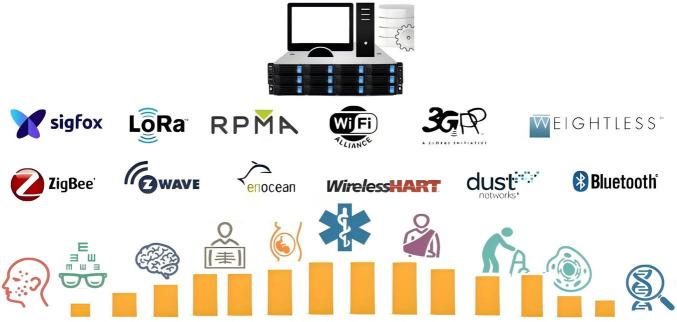
Major communication protocols in IoT technology.

Wireless communication technologies are integral to IoT applications in smart agriculture, smart homes, smart cities, and smart healthcare. Various wireless communication technologies, such as 5G, WiFi, ZigBee, LoRa, NB-IoT, and Bluetooth, are widely used in IoT systems as shown in Figure 1.

#### 2.2.1 High-speed wireless communication networks

High-speed wireless communication networks are crucial for optimizing the interconnectivity of medical devices and improving healthcare services and supply chain management. Srinivasu et al. (36) proposed a 6G-driven networking framework for healthcare applications, enabling fast and efficient communication among medical devices for smart diagnostics, patient-centered treatments, and remote monitoring. Donghua et al. (37) introduced a novel cross-ring neural network model for image compression and security in wireless body area networks (WBANs), demonstrating efficient high-speed data processing capabilities. Sodhro et al. (38) proposed an adaptive QoS (Quality of Service) computation algorithm for efficient monitoring of medical data processing, enhancing network performance in medical applications. Donghua et al. (37) introduced a cross-ring neural network for image compression and security in WBANs, demonstrating efficient data processing and resistance against attacks. These studies collectively highlight the importance of high-speed network connections in optimizing medical device interconnectivity to improve healthcare services and supply chain management.

#### 2.2.2 Low-power wireless communication protocols

Low-power wireless communication protocols, such as ZigBee and Bluetooth Low Energy (BLE), are used for medical device communication, particularly suitable for wireless wearable sensor systems due to their low energy consumption and good network range. Rehman et al. (39) proposed a reliable bidirectional data transmission method using ZigBee for secure medical IoT, enhancing network performance and security for effective communication among medical devices. Rehman et al. (40) illustrated that ZigBee technology can enable efficient communication among medical devices but requires enhanced security measures to prevent various network attacks. Padma and Babu (41) achieved efficient and secure communication in ZigBee networks using DNA sequence encryption, optimizing encryption for limited computational resources and enhancing communication security among medical devices. Yu et al. (42) combined topology structure and hybrid routing strategies to optimize load balancing in BLE networks, improving communication efficiency among medical devices compared to traditional methods like ZigBee. Wang et al. (43) studied a novel CTC protocol designed with pseudo-random sequences for low-cost direct communication between WiFi and ZigBee in noisy indoor environments, achieving synchronization times less than 0.5 ms and accuracy greater than 84% at channel occupancy rates up to 50%. Sriram (44) developed a low-power BLE protocol enabling efficient communication among medical devices in wireless sensor networks (WSNs) with a maximum data transfer rate of 8.4 Mbps and extended communication ranges up to 400 meters. Kozhubaev et al. (45) developed BLE5.0 technology supporting multi-node data collection, high-speed transmission, and low power consumption, enabling efficient communication among medical devices suitable for wearable medical applications.

### 2.3 Edge computing and cloud computing

Edge computing and cloud computing play complementary roles in IoT systems. Edge computing processes data near the source or at local nodes, reducing latency and bandwidth requirements, making it suitable for real-time and latency-sensitive tasks. Cloud computing provides powerful centralized processing and storage capabilities, ideal for large-scale data analysis and long-term storage. Combining both approaches allows IoT systems to achieve efficient data processing and transmission while maintaining system flexibility and scalability.

#### 2.3.1 Edge computing

Edge computing enhances real-time and sensitive tasks in IoT systems. Verma and Fatima (46) explored how edge computing enhances smart healthcare applications through real-time analysis, highlighting the benefits of reduced latency and improved data privacy, enabling healthcare providers to respond faster and more effectively to patient needs. Ghadi et al. (47) discussed the integration of mobile edge computing with 5G technology in healthcare services, exploring how this integration improves service responsiveness and efficiency, providing faster and safer patient data processing and analysis. Hemmati et al. (48) systematically reviewed the implementation of edge artificial intelligence for healthcare big data, focusing on real-time activity recognition and patient monitoring. They emphasized the importance of edge computing in reducing latency and accelerating the processing of medical data. Urblik et al. (49) proposed a modular framework for edge computing that supports real-time data processing and analysis in healthcare. The study highlighted how edge computing reduces latency and bandwidth usage, allowing for more direct and effective responses to patient data. Verma and Fatima (46) reviewed how edge computing improves the security and reduces latency of IoT healthcare systems by processing and analyzing data near the source, avoiding the need to transmit all data to a central cloud. These studies collectively illustrate the crucial role of edge computing in enhancing the real-time responsiveness of healthcare systems, enabling better and faster patient care.

Although edge computing brings numerous advantages, it also entails certain limitations. Firstly, edge devices typically have limited computational capabilities and storage capacity, which may constrain the execution of complex data analysis tasks. Secondly, since edge computing necessitates maintaining software updates and service consistency across a distributed network, it adds to the management complexity. Lastly, the widespread adoption of edge computing could lead to increased investment in hardware infrastructure, including the deployment costs of edge servers and gateway devices. In summary, while edge computing enhances the real-time responsiveness of healthcare systems, it is also imperative to overcome these technical challenges.

#### 2.3.2 Cloud computing

Cloud computing plays a key role in managing large volumes of medical data and providing flexible services for the healthcare industry. Cloud technology integrated with artificial intelligence and distributed data analysis models improves the efficiency of managing electronic health records, ensuring continuous access to personalized healthcare information. Lakhani (50) reviewed how cloud integration in healthcare can handle massive medical data and provide flexible services, enhancing patient care, operational efficiency, and the overall potential of healthcare delivery. Himanshu and Punhani (51) illustrated how cloud computing provides flexibility, scalability, and cost-effectiveness for managing healthcare data, enabling personalized medicine and predictive analytics. Dhote et al. (52) demonstrated how cloud computing efficiently processes large volumes of medical data through Distributed Data Analysis and Organizational Models (DDAOM), enabling flexible service provision and minimizing errors in mobile healthcare systems. Praveen et al. (53) introduced a novel approach, the DACAR platform, ensuring secure storage and processing of sensitive medical data in the cloud, enhancing fusion-based healthcare systems with scalability and precision. Zhang et al. (54) explained how the integration of cloud computing with advanced algorithms, such as the Normal Distribution Method and Incremental Higher Order Possibility C-Means Algorithm, enhances the processing of large medical datasets, ensuring efficient data segmentation, processing, and clustering across multiple hospitals.

Although cloud computing offers numerous benefits, it also presents certain limitations. Firstly, reliance on internet connectivity means that service quality can be compromised under unstable network conditions. Secondly, data privacy and security represent major challenges for cloud computing, particularly when dealing with sensitive medical information. While encryption and other security measures can mitigate these concerns to a degree, completely eliminating risks remains virtually impossible. Additionally, due to the sensitivity of medical data, compliance and regulatory requirements may restrict the application of cloud computing in certain regions.

### 2.4 AI and big data analytics

**FIGURE 2:**
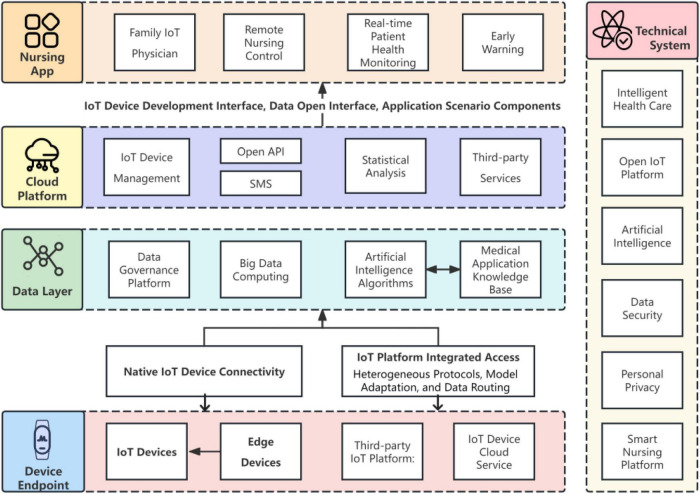
AI and big data-driven medical IoT for enhanced diagnostics and personalized healthcare.

AI and big data analytics play pivotal roles in medical IoT, enhancing diagnostic accuracy, personalizing treatment plans, and predicting disease trends. In the domain of medical IoT as shown in Figure 2, artificial intelligence and big data analytics serve as transformative agents, significantly enhancing diagnostic accuracy, personalizing treatment strategies, and forecasting disease patterns with remarkable precision. The process initiates with the collection of comprehensive training datasets meticulously sourced from various repositories, including electronic health records (EHRs), wearable devices, and patient surveys. These data are then subjected to an intensive training regimen, wherein sophisticated algorithms are trained to discern patterns and establish correlations within the information.

Once the training phase is complete, the AI model becomes a potent instrument for healthcare professionals. In operational mode, the model receives real-time inputs, processing them through a structured decision-making framework. This reconstructed model generates predictive outputs that facilitate early disease detection, personalized treatment recommendations, and forecast future disease trends. The entire process underscores the potential of AI and big data analytics to revolutionize healthcare delivery and improve patient outcomes.

AI-driven clinical decision support systems (CDSS) analyze vast amounts of medical data to predict disease trends, personalize treatment plans, and improve diagnostic accuracy. Prajapati and Prajapati (55) reviewed various AI-driven CDSS, emphasizing their applications in diagnosis and treatment planning across different medical fields. Ferdush et al. (56) surveyed the role of AI language models, such as ChatGPT, in clinical decision support, including their applications in diagnosis and treatment planning, and the challenges of integrating these systems into clinical practice. Demuth et al. (57) studied an AI-based decision support system called StrokeCopilot aimed at aiding the treatment of acute ischemic stroke, improving the timeliness and outcomes of treatment. Eisenstein (58) discussed Shmulevich’s group’s digital twin project for acute myeloid leukemia, using clinical data to predict drug responses and avoid side effects, with a focus on real-world data to enhance personalized treatment AI models. Savage (59) examined how AI uses deep learning algorithms to integrate diverse data types (imaging, clinical indicators, genetic testing) to improve diagnostic accuracy. The combination of different data modalities enhances AI’s ability to make precise diagnoses. Mittermaier et al. (60) explored the collaboration between AI systems and physicians to improve clinical decision-making, particularly in routine care settings, highlighting the challenges and strategies for effective implementation. Luchini et al. (61) highlighted how advances in machine learning help oncologists determine the best treatment strategies for cancer patients by predicting drug responses and avoiding side effects through the analysis of clinical data. Lenharo (62) summarized how AI integrates data from various sources, such as imaging, clinical indicators, and genetic testing, to improve diagnostic accuracy and overall diagnostic processes. Real-time data collected by smart sensors and devices facilitates remote monitoring and immediate medical decision-making, improving patient care and health management.

Despite the numerous advantages of AI and big data analytics in healthcare, such as improved diagnostic accuracy and personalized treatments, these technologies also come with certain limitations. Firstly, the quality and integrity of data are paramount for the effectiveness of AI models; erroneous or incomplete input data can lead to incorrect diagnostic outcomes. Secondly, privacy and ethical concerns continue to pose barriers to the application of AI technologies in the medical field. Additionally, the lack of transparency and explainability in AI systems may hinder the trust of both medical professionals and patients. Finally, the widespread adoption of AI technologies requires ongoing training and support to ensure that healthcare professionals can properly understand and utilize the information provided by these technologies.

### 2.5 Privacy and security in electronic health records

One fundamental challenge in using electronic health records (EHRs) in healthcare is protecting patient privacy. Kiania et al. (63) reviewed how blockchain technology enhances integrity, privacy, and security, facilitating rapid action by experts and health organizations. Through smart contracts, a secure and effective infrastructure can be established, improving healthcare quality and individual wellbeing. Wu et al. (64) proposed the use of blockchain technology for securing privacy information in healthcare systems, with storage response times kept below 1,000 ms and maximum information throughput reaching 550 kbit/s, utilizing elliptic curve Diffie-Hellman keys to provide effective protection of information. Zarour et al. (65) designed a hybrid fuzzy-ANP-TOPSIS method to evaluate the impact of blockchain technology models on maintaining the security of EHRs. Zhang et al. (66) proposed a 5G-integrated PTBM scheme capable of tracking patients and close contacts while protecting patient information. Zhang et al. (67) introduced a medical data sharing scheme based on consortium blockchain, strengthening key management to protect patient privacy. Alsayegh et al. (68) combined private blockchain with smart contracts on a consortium blockchain to encrypt and securely store and read medical personal information. Srinivasu et al. (36) used a knapsack-based system with a knapsack greedy algorithm to encrypt and decrypt medical brain tumor data, achieving shorter computational times (the proposed blockchain-based knapsack method required 68.81 s to encrypt 100 data blocks).

## 3 Smart IoT nursing platform framework

### 3.1 Overview of platform architecture

**FIGURE 3:**
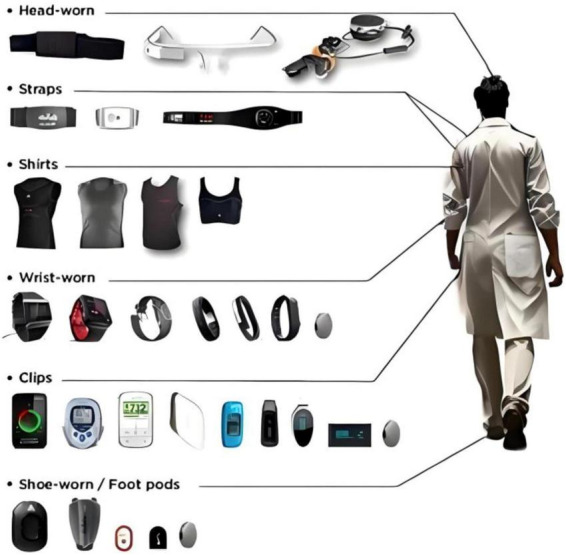
Smart IoT nursing platform framework.

The architecture of a smart Internet of Things (IoT) nursing platform comprises four primary layers: the perception layer, the network layer, the platform layer, and the application layer. The design principles include modularity for scalability, data security and privacy protection, high efficiency with low power consumption, interoperability and standardization, as well as user-friendliness and ease of use (69, 70). These principles ensure the stability, flexibility, and maintainability of the platform, providing support for applications in smart cities, smart manufacturing, and more. The overall architecture of the intelligent nursing platform based on IoT smart sensing settings is illustrated in Figure 3.

The intelligent Internet of Things (IoT) nursing platform adopts a multi-layered architecture design encompassing device terminal layers, data layers, cloud platform layers, nursing application layers, and integration with existing medical systems. The device terminal layer integrates a variety of IoT sensing devices and actuators, including wearable health monitoring devices, smart home appliances, and medical instruments, complemented by edge computing nodes for primary data processing and anomaly detection. The data layer relies on distributed storage systems and big data processing frameworks to achieve efficient storage and preprocessing of massive datasets, applying advanced machine learning algorithms for pattern recognition and predictive analytics. As the core of the platform, the cloud platform layer not only provides support for device management and cross-system communication but also integrates business intelligence tools to facilitate the visualization of data analysis, supporting third-party service integration to enhance platform functionality. The nursing application layer is designed for end-users, offering real-time health monitoring, early warning, and nursing management functionalities via an intuitive human-machine interaction interface. At the level of integration with existing medical systems, standardized communication protocols such as HL7 and FHIR, along with health information exchange mechanisms, are implemented to ensure interoperability with traditional healthcare information systems, strictly adhering to regulatory requirements such as HIPAA to safeguard data security and privacy protection.

Successful implementations of smart nursing platforms have been reported. Zhou et al. (71) described the successful deployment of an intelligent health management system in a nursing home, utilizing IoT, deep learning, and cloud computing to continuously monitor and intervene early in elderly healthcare. Wen et al. (72) demonstrated that a Smart Patient Care System (SPCS) had been successfully implemented in medical centers, reducing nurse response times, decreasing false alarms, and enhancing care efficiency and quality. Boo and Oh (73) surveyed a pilot project in South Korea that employed AI-enhanced IoT for elderly healthcare, finding increased customer satisfaction, improved health behaviors, and governmental support as key factors contributing to the success of such initiatives. These case studies underscore the potential benefits of smart nursing platforms in enhancing healthcare services, improving patient outcomes, and optimizing nursing workflows.

### 3.2 Key IoT components and functions

Key components of IoT systems include sensors and actuators (for data acquisition and command execution), network communication modules (ensuring device connectivity), gateways (processing and transmitting data), IoT platforms (managing devices and analyzing data), and security components (protecting data and devices from threats). Functionally, IoT systems enable real-time monitoring, data analysis, remote control, and automation across various domains including smart cities, industrial IoT, and smart homes.

#### 3.2.1 Sensor deployment strategies for real-time monitoring modules

**FIGURE 4:**
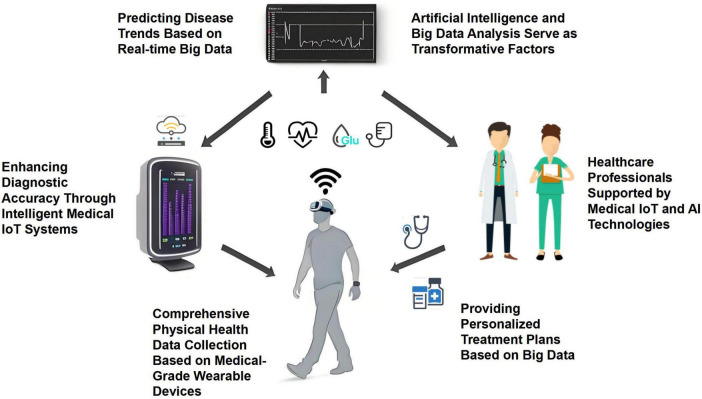
IoT wearable devices for vital sign monitoring and other data collection.

Strategies for deploying sensors in real-time monitoring modules are critical across different scenarios. Figure 4 illustrates various IoT wearable devices placed at different body locations and serving specific functions for collecting vital signs and other types of data.

Sundas et al. (74) reviewed hierarchical architectures for IoT that convert sensor data into clinical inputs, encompassing sensing, transmission, processing, storage, mining, and learning to enhance real-time medical monitoring in IoT applications. Liang et al. (75) proposed two algorithms based on sub-module functional optimization to maximize surveillance capabilities using mobile sensors in large pipeline networks. Jiang et al. (76) introduced an enhanced binary particle swarm optimization (MBPSO) strategy to determine the minimum number of sensors required for effective real-time monitoring in grid systems, achieving efficient monitoring of over 89.6% of conductor temperature high events with a root mean square error of less than 0.8°C in reconstructed conductor temperature distributions. Wang et al. (77) developed a graph flow-based algorithm for intelligent sensor network deployment offset detection to optimize sensor node updates for efficient monitoring. Rosa et al. (78) presented a system that uses various sensors, such as electrocardiograms, temperature, and motion sensors, for disease analysis and continuous monitoring, with data transmission accuracy rates exceeding 95%. Balakrishnan and Ranganayaki (79) introduced a wireless health monitoring system that employs sensors for real-time patient data collection, transmission, and alerts, enhancing healthcare monitoring efficiency and patient safety. Liu et al. (80) discussed the use of low-cost sensors in healthcare monitoring with wireless communication (77%) and algorithms such as SA, SVM, and KNN. Harb et al. (81) proposed a sensor-based data analysis system for real-time patient monitoring in healthcare, focusing on emergency detection, sensing frequency adaptation, and patient condition prediction. Shafi et al. (82) proposed an IoT-based patient health monitoring system that uses sensors for real-time data collection, transmission to cloud storage, and remote access by healthcare professionals. These sensor deployment strategies not only enhance patient care but also assist healthcare professionals in making critical decisions based on accurate and timely information.

#### 3.2.2 Application of AI algorithms in disease management

In disease management, artificial intelligence (AI) algorithms are increasingly used in analytics and predictive modules. These algorithms, such as machine learning models, have shown promise in optimizing treatment strategies and improving patient outcomes in chronic conditions like diabetes.

Pandey et al. (83) demonstrated that AI algorithms predicted tuberculosis progression with 87.5% accuracy and 88.2% sensitivity, showcasing the potential of AI to transform disease management through personalized treatment plans based on clinical data. Grampurohit and Sagarnal (84) utilized AI algorithms such as random forests, SVM, and Naïve Bayes for disease prediction, achieving adjusted random forest accuracy rates of 90%, demonstrating the potential of AI in disease management. Cho et al. (85) developed an ML-based predictive model to assess participants’ five-year risk of atherosclerotic cardiovascular disease, showing moderate to good discrimination (C-statistic 0.70–0.80). Glass et al. (86) used Keras for AI modeling and training, where AI algorithms successfully distinguished myocyte injury (1R2 grade) from non-myocyte injury (1R1A grade) with 94% validation accuracy. ACR and healing injury identification accuracy was 98–99%, while normal myocardial cell identification accuracy was 99%. Yu (87) analyzed multiple indicators using eight different algorithms based on the UCI dataset, achieving the highest accuracy and reliability in predicting heart disease. Tarumi et al. (88) utilized AI algorithms for predictive analysis, decision support system reinforcement, and integration with electronic health records to improve patient outcomes in chronic disease care. Isiaka et al. (89) illustrated the integration of AI into infectious disease surveillance, enabling early detection, outbreak management, and proactive resource allocation, thereby strengthening public health responses and ensuring community welfare. Farrag et al. (90) detailed AI algorithms in chronic hepatitis C virus management, including the prediction of new therapies, prioritizing patients for antiviral drugs, and outperforming traditional diagnostic tools in detecting infections and related diseases. Zhuhadar and Lytras (91) described AutoML technology in diabetes diagnosis, utilizing AI algorithms for predictive modeling and identifying risk factors such as glucose levels, body mass index, diabetes pedigree function, and blood pressure, thereby reinforcing disease management in healthcare. Kargbo (92) detailed AI algorithms in disease management that analyze biomarker data for health predictions, enabling early intervention and personalized treatment plans.

These cases demonstrate the potential of AI to revolutionize clinical decision-making, personalize treatment plans, and optimize interventions to improve patient outcomes and public health results.

#### 3.2.3 Design and case studies of automated response mechanisms

Automated response mechanisms in healthcare are designed to leverage AI and machine learning technologies to automatically identify emergencies through real-time patient data monitoring and provide timely interventions. These systems integrate sensors, data analysis tools, and alert systems to ensure rapid response by caregivers, optimizing therapeutic effects (93, 94). Automated response mechanisms also include personalized care plans and intelligent reminders, enhancing patient safety and care quality (95). In this way, healthcare can achieve higher efficiency and accuracy. Ni et al. (96) used automatic search relevance assessment in healthcare environments, highlighting the role of infrared technology in improving search engine performance. Jahanshahi et al. (97) proposed an intelligent auto-reply to generation system for medical chat services, achieving 83.28% accuracy through historical doctor-patient message analysis and machine learning algorithm training. Bagattini et al. (98) studied the implementation of an automated system in a central pharmacy at a private hospital in Latin America, resulting in reduced dispensing errors down to 0.018%, indicating improved pharmacy management. These automated systems are critical in major transformations within global healthcare institutions, optimizing resources and processes.

### 3.3 Security and privacy protection strategies

Security and privacy protection strategies in IoT-enabled healthcare encompass several critical aspects, including the adoption of encryption technologies to secure data transmissions and ensure secure communications between devices, the implementation of strict access controls to limit data access rights, regular security assessments and vulnerability patching to prevent cyberattacks, the utilization of data anonymization techniques to protect patient privacy, and the establishment and adherence to relevant privacy protection laws and standards to ensure compliance in data handling.

Recent advances in data security technologies in the healthcare sector have witnessed the emergence of innovative methods. Odera (99) highlights recent data security technologies including digital image encryption, steganography, biometrics, rule-based policies, prescriptive analytics, blockchain, cloud security, MapReduce, machine learning algorithms, and anonymization techniques. Madavarapu et al. (100) introduced an Advanced Integrated Data Security (AIDS) framework for cloud healthcare systems, combining encryption, access control, anomaly detection, and intrusion prevention to enhance data security. Basha (101) enhanced the security of healthcare data using quantum cryptography, providing robust encryption that surpasses AES in terms of both efficiency and confidentiality (quantum cryptography requires 352,237 milliseconds, compared to 310,285 milliseconds for AES), ensuring secure storage and regulatory compliance of patient information. Khan et al. (102) used an adaptive hybrid encryption and steganography model to strengthen data security in the healthcare industry by encrypting and hiding diagnostic text data within medical images, ensuring secure transmission through the Internet of Healthcare Things (IoHT). Abdelaziz and Alia (103) proposed an integrated artificial intelligence framework for information security and management, leveraging AI algorithms for secure automated transactions to enhance data security in healthcare systems. These technologies address vulnerabilities caused by data breaches and unauthorized access, ensuring the confidentiality, integrity, and privacy of sensitive medical data stored and processed in cloud environments.

As the digitization of medical information continues to increase, legal requirements for data usage and privacy protection in healthcare become paramount. Countries such as Russia and India are focusing on strengthening their data protection frameworks to safeguard patient privacy (104, 105). In Europe, the use of patient information is subject to numerous legal and ethical obligations, with the General Data Protection Regulation (GDPR) emphasizing explicit consent for data processing, which contrasts with common law practices (106, 107). Although informed consent plays a crucial role in data handling, the obligation to share data to improve healthcare may conflict with existing privacy laws and regulations (108). The integrity, availability, and confidentiality of healthcare information are essential, necessitating a comprehensive approach to data protection within the evolving landscape of healthcare data processing.

## 4 IoT Implementation case studies in healthcare

### 4.1 Global application of IoT in nursing

Comparative analyses of smart nursing platforms in developed countries reveal varying levels of technological integration and development, influenced by historical, geographical, political, and professional factors (109). In Western countries, blogs have been integrated into nursing education to enhance learning outcomes, while Eastern countries are still exploring the potential of this technology in nursing education (110). De Raeve et al. (111) found that out of 35 European countries surveyed, 11 have national legislation specifying minimum educational requirements for nurses, with most requiring a master’s degree for advanced practice nurses. Additionally, Estonia, Iceland, Malta, and Sweden mandate at least two years of practical experience (112). The use of mobile health systems, particularly in China, has facilitated the development of Java Web-based intelligent outpatient care service platforms to address evolving healthcare needs (67, 113). In some advanced regions of China, 5G networks and artificial intelligence robots have been deployed in smart nursing platforms, enhancing personalized medical services and accelerating digital transformation in healthcare (114). In the United States, emphasis is placed on integrating smart nursing platforms into home care delivery processes to improve care quality and reduce costs, as evidenced by studies on the integration of Smart Care Platforms (SCPs) into home care provision (115, 116). Perspectives from Italy, Brazil, and the Philippines highlight the integration of clinical nursing information systems with electronic health records and the challenges and advancements in nursing informatics across different healthcare systems (117).

The implementation of Internet of Things (IoT) technologies in healthcare settings in low- and middle-income countries (LMICs) has been a topic of interest in recent literature. (118) conducted a multi-country case study focusing on the adoption and implementation of a network of university hubs for social innovation in healthcare delivery across Africa, Asia, and Latin America. The study aimed to describe the model, components, and implementation process of these hubs, identifying enablers and barriers that could be relevant to other LMIC universities interested in social innovation. In the context of the COVID-19 pandemic, (119) highlighted the importance of early implementation of effective measures by governments in controlling the spread of the virus, particularly in LMICs. The study emphasized that despite wealthier countries having elevated healthcare capacity, early and incremental government actions are crucial in LMICs to prevent the rapid spread of the virus. (120) focused on the safe handling of chemotherapy drugs in LMIC healthcare facilities, conducting a survey among 53 facilities in different income categories. The study provided an overview of practices in dealing with chemotherapy drugs, shedding light on the challenges faced by healthcare facilities in LMICs. Furthermore, (121) examined national hospital accreditation programs in the Eastern Mediterranean Region, including case studies from Egypt, Jordan, and Lebanon. The study addressed design and implementation issues of these programs, offering insights into the healthcare quality improvement initiatives in LMICs. In a study by Dkhimi et al. (122), the authors synthesized findings from six country case studies examining multiple funding flows to public healthcare providers in LMICs. The qualitative findings revealed the potential impact of multiple payment streams on healthcare provider behavior, highlighting the complexities of funding mechanisms in LMIC healthcare systems. Overall, the literature review indicates a growing interest in exploring the implementation of healthcare initiatives, such as social innovation hubs, COVID-19 control measures, chemotherapy drug handling practices, hospital accreditation programs, and funding flows in LMICs. These studies provide valuable insights into the challenges and opportunities for implementing healthcare interventions in resource-constrained settings.

### 4.2 Specific effects of IoT on enhancing nursing levels globally

Abdulrazak et al. (123) utilized environmental sensors, enhanced device monitoring, and change-point detection algorithms to study urinary function in 16 French patients, focusing on urinary tract infections and irritable bowel syndrome. The algorithm analyzed daily bathroom frequency and activity changes, enabling healthcare providers to track health status through alerts, thereby improving diagnostic and therapeutic outcomes. Yesmin et al. (124) demonstrated that IoT interventions in healthcare improved hand hygiene compliance rates and staff experiences, with a direct reduction in patient falls leading to improved nursing times. Abril-Jiménez et al. (125) found that digital home hospitalization services based on IoT enhanced patient autonomy, reduced hospital stays (from an average of 11 to 6 days), implemented four disease-specific protocols with up to 16 different pathways, lowered readmission rates, achieved 95% patient satisfaction, and increased professional trust and workload management. Mudawi (126) proposed an IoT-based intelligent ICU patient monitoring system capable of real-time measurement of patient parameters, greatly assisting doctors and hospitals in continuous patient monitoring and rapid decision-making. Yongjoh et al. (127) implemented the NU Medical system using blockchain technology in a Thai hospital with 157 healthcare workers, achieving rapid and confidential access to medical conditions, enhancing staff efficiency and patient satisfaction.

### 4.3 Case analysis of IoT enhancing nursing levels in China

Ni et al. (128) used telemedicine technology to promote a renal anemia management system (the Red China project aimed to develop a dialysis registration system based on the WeChat mobile platform) which resulted in a decrease in the proportion of patients with hemoglobin levels < 8 g/dL from 4.96 to 4.08%. Wang and Liu (129) designed the RASAP-POCT system utilizing IoT to support real-time data collection, on-site screening, and integration with contact tracing systems, providing saliva sample test results within 20–30 min with lower reagent complexity (approximately 50% less per reaction) and cost, while maintaining higher reliability, thus improving public health surveillance and intervention and enhancing SARS-CoV-2 testing in China. Shen et al. (130) described China’s “no second visit” program, which leverages internet-based care delivery methods to enhance outpatient services, reducing waiting times (appointment waiting time shortened by 4.05 min; consultation time shortened by 0.37 min; payment waiting time reduced by 3.09 min), increasing efficiency, and improving patient satisfaction. Qin et al. (131) developed an IoT cloud computing platform with an IN model, where wireless temperature monitoring systems (WTMS) and vital sign monitoring systems (VSMS) play a crucial role. Real-time monitoring of patients through radiofrequency identification of sensors attached under the armpit significantly improved the efficacy of HGG treatment for pediatric pneumonia sepsis and reduced adverse reactions. Hu et al. (132) found that implementing a tertiary enteral nutrition care system under the “internet + healthcare” model has been shown to improve nurses’ cognition, behavior, and core competencies in enteral nutrition safety care (*P* < 0.05), leading to positive clinical outcomes.

### 4.4 Technological efficacy and challenges

The application of the Internet of Things (IoT) in healthcare has expanded considerably, bringing about significant technological benefits and challenges. Through real-time monitoring and data collection from sensors and devices, IoT enables continuous monitoring of patients’ physiological indicators, enhancing the precision of diagnostics and treatments. Additionally, the proliferation of remote healthcare and nursing services provides convenience for patients in remote areas and those with mobility issues, markedly improving healthcare efficiency.

However, the quality and reliability of data pose significant challenges to nursing decisions. The accuracy and completeness of data directly impact the reliability of medical decisions (133). Bernardi et al. (134) argue that well-established and validated workflows for healthcare data quality assurance are beneficial for ensuring data quality. Poor dimensions and outcomes of data quality can be evaluated through various classification methods (135). Elucidating the intricate relationships among DQ dimensions and their impacts on clinical, clinician, research, business process, and organizational outcomes. It identified six core dimensions of DQ—accessibility, accuracy, completeness, consistency, contextual validity, and currency—that collectively form the foundation of data quality. Notably, consistency was recognized as a pivotal factor influencing all other dimensions, while both consistency and accessibility exert significant influence on all DQ outcomes. To address these DQ issues, we propose a set of solutions: Firstly, there is a need to enhance the management of data consistency to ensure uniformity across different data sources, thereby mitigating errors arising from inconsistencies. Secondly, optimizing data accessibility is essential, ensuring authorized users can efficiently obtain necessary information, which in turn enhances the effectiveness of clinical decision-making. Lastly, it is imperative to establish a continuous DQ monitoring mechanism to periodically evaluate the status of each DQ dimension and adjust data governance strategies accordingly to maintain high standards of data quality. This framework provides healthcare institutions with a systematic approach to addressing DQ issues, aiding in the optimization of resource allocation and the enhancement of service quality and efficiency.

Data interoperability is a significant challenge in modern digital environments, involving the ability to share and exchange data among multiple systems or platforms. However, in practice, data interoperability faces numerous issues such as incompatible data formats, insufficient data standardization, data security concerns, and lack of common data interfaces and protocols. These problems not only limit the value and utilization efficiency of data but may also have negative impacts on business decision-making and strategic planning.

To address these data interoperability issues, a series of effective measures should be taken. First, unified data standards and norms should be established to ensure consistency and interpretability of data. Second, data security and privacy protection measures should be strengthened by developing corresponding policies and regulations to guarantee the safety and legal use of data. Additionally, common data interfaces and protocols can be developed so that different systems or platforms can communicate and interact with each other. Simultaneously, promoting open-source technologies and tools can enhance data openness and sharing, improving data utilization and value. Finally, establishing cross-domain and cross-industry cooperation mechanisms can facilitate data resource integration and sharing, driving innovation and development of data (136).

With the advent of the digital era, issues related to data security and privacy breaches have become increasingly severe. Unauthorized access, modification or destruction of data can lead to loss, leakage or malicious use of data, which not only poses a threat to individual privacy but also may have serious implications for business operations and national security. To address these problems, it is necessary to establish sound management systems and technical safeguards, enhance employee awareness training, introduce legal and policy regulation, promote privacy protection technologies and products, and raise public awareness and self-protection capabilities. Only by continuously strengthening management and technological innovation can effective protection of data security and personal privacy be achieved (136).

User acceptance and adherence represent another critical challenge, especially for elderly individuals and those unfamiliar with technology. Resistance to and difficulties in using IoT devices affect their adoption and effectiveness (137). Personalized and intelligent nursing services provided by IoT can increase acceptance among older adults (138). Furthermore, healthcare professionals can improve their acceptance of IoT-based smart healthcare through cognitive and emotional pathways (139).

However, there is also a need to address ethical dilemmas associated with the implementation of IoT technologies in healthcare settings. One such dilemma is the potential deskilling of medical practitioners due to reliance on automated systems. While these systems aim to enhance diagnostic accuracy and patient monitoring, they could inadvertently reduce the clinical skills of healthcare workers if over-reliance occurs (140). Another ethical issue pertains to data ownership and privacy concerns. With vast amounts of sensitive health data being collected, ensuring secure storage and transparent data sharing practices becomes paramount (141).

To mitigate these challenges, robust training programs and regulatory frameworks are essential. Training initiatives should focus not only on technical proficiency but also on ethical considerations, helping both patients and healthcare providers understand the benefits and limitations of IoT applications (142, 143). Regulatory bodies must establish guidelines that protect patient data while promoting innovation and interoperability within the healthcare sector.

Moreover, the high initial investment and ongoing maintenance and update costs of IoT devices pose significant challenges to economic feasibility and sustained development (141). While the initial investment in IoT devices may be high, integrating these technologies into smart city initiatives can facilitate transitions toward greater sustainability (142, 143). Leveraging IoT and digital economy opportunities can promote national development, particularly during crises such as the COVID-19 pandemic, demonstrating the potential for economic growth and resilience through smart education, smart cities, smart homes, and e-health services (140).

Short-term gains include reduced equipment downtime through predictive maintenance, optimized inventory management that minimizes waste, and enhanced staff efficiency via real-time tracking systems. For instance, the University of Washington Medical Center saw a reduction in equipment failure rates and maintenance costs after implementing IoT-based predictive maintenance programs.

Long-term financial implications are even more profound. IoT facilitates preventive care and chronic disease management, potentially decreasing hospital readmissions and treatment costs. Additionally, IoT opens up new revenue streams through telemedicine and personalized health plans. Teladoc Health, for example, expanded its telehealth offerings using IoT technology, boosting profitability.

Case studies illustrate these benefits clearly. The Royal Adelaide Hospital’s use of IoT for asset tracking and environmental monitoring led to better asset utilization and patient safety. Philips Healthcare’s partnership with hospitals to deploy IoT-enabled patient monitoring systems demonstrated the ability to detect issues proactively, reducing complications and associated costs.

The integration of IoT technologies in nursing practice can significantly mitigate the impact of workforce shortages. By leveraging smart devices and sensors, IoT enables remote patient monitoring, which reduces the need for constant on-site staffing and allows healthcare professionals to manage a larger number of patients efficiently. For instance, smart wearables that monitor vital signs can transmit real-time data to nursing stations, enabling staff to prioritize care and intervene promptly when necessary (144). Additionally, the use of telehealth platforms facilitated by IoT can extend the reach of healthcare providers, offering virtual consultations and reducing the physical demands on the nursing workforce (26).

IoT has the potential to revolutionize patient monitoring in rural areas, where access to healthcare services is often limited. By deploying IoT-enabled sensors and devices, remote health monitoring becomes feasible, allowing patients in rural settings to receive continuous care without the need for frequent travel to urban medical centers. This is particularly beneficial for patients with chronic conditions who require regular health check-ups and medication management (145). Furthermore, IoT can facilitate the collection of health data from dispersed locations, providing healthcare providers with a comprehensive view of community health trends and enabling more informed care decisions (113).

Thus, while initial IoT investments may be high, the long-term financial viability and operational improvements make IoT solutions indispensable for modern healthcare providers seeking sustainable growth and enhanced patient care.

## 5 Discussion and future direction

### 5.1 Technological trends

The application of Internet of Things (IoT) technology in healthcare is rapidly advancing, with innovations primarily focused on novel sensors, device technologies, and the integration of artificial intelligence (AI).

Wearable and implantable sensors enable real-time monitoring of patients’ physiological data (146, 147), while smart medical devices such as surgical robots and telemedicine equipment enhance the precision and efficiency of medical procedures (148, 149). Supported by 5G technology, the transmission speed and stability of telemedicine devices have improved significantly, enabling physicians to provide more convenient diagnostic and treatment services (150).

Large models and AI play a critical role in medical data analysis, personalized medicine, and intelligent diagnostic systems (151, 152). AI can process large volumes of medical data, identify patterns of diseases, predict disease progression, and offer personalized treatment plans (153, 154). Furthermore, the integration of IoT platforms allows for unified management of data from various devices and sensors, further enhancing the operational efficiency and service quality of healthcare institutions (155). In the future, the application of IoT in healthcare will evolve toward greater intelligence, precision, and personalization, driven by the convergence of novel sensors, advanced device technologies, and AI, to comprehensively elevate the level and efficiency of medical services.

### 5.2 Policy recommendations and collaborative models

The application of IoT technology in healthcare has become a global focal point, making international cooperation and resource sharing particularly important. Through cross-border collaboration, countries can share medical technologies, devices, and data, leveraging each other’s strengths. For example, the United States and the European Union have a lead in medical device and sensor technologies (156), while Asian countries excel in big data analytics and AI applications (157). Cross-border cooperation facilitates the development of advanced IoT healthcare solutions and promotes the standardization of global medical data, enhancing interoperability and availability of medical information (17). Additionally, resource sharing optimizes global allocation of healthcare resources, allowing remote regions to consult with experts in major cities through telemedicine platforms, reducing misdiagnosis and treatment costs (158). This was particularly evident during the COVID-19 pandemic in 2019, when governments and health experts implemented various preventive measures and collaborated on resource sharing to curb the spread of the virus (159).

The development trend of IoT applications and technologies in the healthcare field is rapidly evolving with the integration of advanced technologies such as artificial intelligence (AI), 5G networks, and blockchain. Ge et al. (160) discuss the potential of 5G networks in supporting mission-critical IoT services, particularly through the implementation of the tactile Internet (TI). This advancement in wireless communication networks enables real-time control communications for IoT applications. Additionally, Wijethilaka et al. (161) highlight the importance of network slicing in realizing IoT applications within 5G networks, thereby addressing technical challenges through this innovative technology.

Artificial intelligence (AI) plays a pivotal role in the healthcare sector. Vaishya et al. (162) and Almaiah et al. (163) underscore the transformative impact of AI applications, which have been instrumental in addressing challenges such as the COVID-19 pandemic and enhancing security and privacy in digital healthcare using IoT platforms. Furthermore, Taimoor et al. (164) emphasize the significance of AI in facilitating personalized healthcare services, illustrating the potential of AI within the modern healthcare Internet of Things (HIoT).

Blockchain technology also plays a vital role in the healthcare sector. Almaiah et al. (163) and Panagiotidis (165) discuss the integration of blockchain in healthcare applications, which ensures data security, privacy, and authentication. Specifically, Panagiotidis (165) examines the educational applications of blockchain, highlighting its features, advantages, and potential challenges within the educational sector.

Overall, the development trend of IoT applications and technologies in the healthcare field is driven by the convergence of AI, 5G, and blockchain technologies. These advancements are transforming the healthcare industry by enabling personalized healthcare services, enhancing data security and privacy, and supporting real-time control communications for mission-critical IoT services. The utilization of these advanced technologies is essential for the paradigm shift toward a distributed, patient-centric approach in healthcare, as emphasized by Abir et al. (166).

The integration of Internet of Things (IoT) technology into nursing practice is gaining increasing significance within the healthcare sector. As elucidated by Al-Rawashdeh et al. (167), key drivers for the adoption of IoT applications in nursing care encompass delivering deep insights and strategic guidance to leaders and system developers in the healthcare industry. This highlights the imperative for collaboration between nursing professionals and IoT technology developers to ensure the seamless and effective implementation of these technologies.

To catalyze this collaboration, healthcare organizations should offer nurses training in artificial intelligence (AI) and opportunities for professional development, as advocated by Yelne et al. (168). Such initiatives are vital for maintaining the currency of nurses’ knowledge and skills, thereby enhancing their capability to collaborate effectively with IoT technology developers. Deep collaboration between nursing professionals and IoT technology developers is essential for the proficient adoption and utilization of IoT applications in nursing care. By fostering a collaborative environment, nurses and developers can ensure that technology is integrated in a manner that not only optimizes patient care but also enhances the overall quality of healthcare outcomes.

In the field of IoT healthcare, regulatory frameworks and standardization policies are crucial for ensuring data security, privacy, and interoperability. Various policy issues influence the integration of big data technologies in healthcare, including data sharing, security, standards, stakeholder engagement, and legal considerations (169). The European legal framework includes the General Data Protection Regulation (GDPR) and the Medical Device Regulation (MDR), addressing data breaches and security measures applicable to IoT medical devices (170). Rathee et al. (171) emphasize the importance of IoMT device standardization for increasing trust and transparency, while Rathee et al. (171) discuss the significance of blockchain technology in securing medical multimedia data. Sadhu et al. (172) highlight the need for standardized regulations and security systems in IoMT due to the risk of exploitation by hackers. Ghosh et al. (173) propose a framework for continuous delivery of IoT services to address latency issues, ensuring service delivery even in areas with poor internet coverage. These insights collectively underscore the critical role of regulations, frameworks, and standards in shaping the future of IoT healthcare.

The collaboration between the European Union (EU) and the United States in the realm of medical device safety standards exemplifies the significant outcomes achieved through concerted efforts to promote consistency between the Medical Device Regulation (MDR) and the US Food and Drug Administration (FDA). This cooperation has not only simplified compliance requirements for businesses but also enhanced global medical device safety standards (174).

In the Asia-Pacific region, the cooperation among APEC member economies, particularly through its Privacy Framework, provides guidelines for cross-border data transfers. This is especially important for IoT healthcare services that rely on real-time data exchange (175).

Initiatives such as the Global Health Data Exchange Initiative aim to establish a global network for sharing health data, fostering knowledge exchange and technical collaboration among countries in health data management. This collaborative effort contributes to the development of unified data protection standards and promotes interoperability in health information systems (176).

## 6 Materials and methods

To enhance the scientific rigor and credibility of our review paper, we have meticulously detailed the systematic processes employed for literature retrieval and screening in the methodology section. Initially, we formulated an exhaustive search strategy targeting major databases such as PubMed, Web of Science, Scopus, and Google Scholar. Keyword combinations included terms like “Internet of Things,” “smart nursing,” “technical challenges,” “international cooperation,” and “policy recommendations,” ensuring the breadth and specificity of the search results. The time span for literature retrieval was set from 2015 to 2024, aiming to capture the latest developments in the application of IoT technologies in healthcare over the past decade.

Subsequently, we established rigorous inclusion and exclusion criteria. Inclusion criteria were strictly defined as English-language articles published in peer-reviewed journals that directly addressed the application of IoT technologies in healthcare, technical challenges, international cooperation, and policy recommendations. Exclusion criteria explicitly ruled out non-peer-reviewed conference abstracts, review articles, editorials, news reports, blog posts, and other documents that were either off-topic or did not contribute substantively to the research subject.

During the screening process, two independent researchers first reviewed the titles and abstracts of all search results to identify potentially eligible studies. Following this, a thorough full-text examination was conducted to further verify compliance with the final inclusion criteria. Throughout the process, any discrepancies were resolved through discussion, ensuring consistency and fairness in the selection of literature.

From the finally included studies, key information including the first author, publication year, study design, main findings, and conclusions were extracted. A narrative synthesis approach was utilized to systematically summarize the research findings and discuss their significance in the context of current IoT applications in healthcare.

## References

[1] Miyashita J Shimizu S Shiraishi R Mori M Okawa K Aita K Culturally adapted consensus definition and action guideline: Japan’s advance care planning. *J Pain Symptom Manage.* (2022) 64:602–13. 10.1016/j.jpainsymman.2022.09.005 36115500

[2] Zou Z Cini K Dong B Ma Y Ma J Burgner D Time trends in cardiovascular disease mortality across the BRICS: an age-period-cohort analysis of key nations with emerging economies using the Global Burden of Disease Study 2017. *Circulation.* (2020) 141:790–9. 10.1161/CIRCULATIONAHA.119.042864 31941371

[3] Xie M Liu X Cao X Guo M Li X. Trends in prevalence and incidence of chronic respiratory diseases from 1990 to 2017. *Respir Res.* (2020) 21:49. 10.1186/s12931-020-1291-8 32046720 PMC7014719

[4] World Health Organization. *Global Health Workforce Statistics Database.* (n.d.). Available online at: https://www.who.int/data/gho/data/themes/topics/health-workforce (accessed July 13, 2024).

[5] Blumenthal D. Wiring the health system–origins and provisions of a new federal program. *N Engl J Med.* (2011) 365:2323–9. 10.1056/NEJMsr1110507 22168647

[6] Liu J Liu S Zheng T Fang J. Development of nursing informatics in Mainland China: a Bibliometric analysis. *Stud Health Technol Inform.* (2021) 284:56–8. 10.3233/SHTI210664 34920470

[7] Exley J Glover R Mccarey M Reed S Ahmed A Vrijhoef H Governing integrated health and social care: an analysis of experiences in three European countries. *Int J Integr Care.* (2024) 24:9. 10.5334/ijic.7610 38344427 PMC10854466

[8] Umer M Aljrees T Karamti H Ishaq A Alsubai S Omar M Heart failure patients monitoring using IoT-based remote monitoring system. *Sci Rep.* (2023) 13:19213. 10.1038/s41598-023-46322-6 37932424 PMC10628138

[9] Alshammari H. The internet of things healthcare monitoring system based on MQTT protocol. *Alexandria Eng J.* (2023) 69:275–87. 10.1016/j.aej.2023.01.065

[10] Astell A Dosanjh S D’Elia T Kokorelias K Stewart S Grigorovich A Personalized tablets for residents in long-term care to support recreation and mitigate isolation. *J Am Med Dir Assoc.* (2024) 25:105022. 10.1016/j.jamda.2024.105022 38763162

[11] Ni Ki C Hosseinian-Far A Daneshkhah A Salari N. Topic modelling in precision medicine with its applications in personalized diabetes management. *Exp. Syst.* (2022) 39:e12774. 10.1111/exsy.12774

[12] Bzai J Alam F Dhafer A Bojoviæ M Altowaijri S Niazi I Machine learning-enabled Internet of Things (IoT): data, applications, and industry perspective. *Electronics.* (2022) 11:2676. 10.3390/electronics11172676

[13] HealthIT.Gov. *Medical practice efficiencies and cost savings.* (n.d.). Available online at: https://www.healthit.gov/topic/health-it-and-health-information-exchange-basics/medical-practice-efficiencies-cost-savings (accessed November 20, 2024).

[14] Kulshreshtha A Kvedar J Goyal A Halpern E Watson A. Use of remote monitoring to improve outcomes in patients with heart failure: a pilot trial. *Int J Telemed Appl.* (2010) 2010:870959. 10.1155/2010/870959 20508741 PMC2874922

[15] Mullins A O’Donnell R Mousa M Rankin D Ben-Meir M Boyd-Skinner C Health outcomes and healthcare efficiencies associated with the use of electronic health records in hospital emergency departments: a systematic review. *J Med Syst.* (2020) 44:200. 10.1007/s10916-020-01660-0 33078276

[16] Paul L Ahmed S Rani T Haque M Roy T Hossain M A smart medicine reminder kit with mobile phone calls and some health monitoring features for senior citizens. *Heliyon.* (2024) 10:e26308. 10.1016/j.heliyon.2024.e26308 38404861 PMC10884519

[17] El-Saleh A Manan Sheikh A Albreem M Honnurvali M. The Internet of Medical Things (IoMT): opportunities and challenges. *Wireless Netw.* (2024) 1–18. 10.1007/s11276-024-03764-8

[18] Huang C Wang J Wang S Zhang Y. Internet of medical things: a systematic review. *Neurocomputing.* (2023) 557:126719. 10.1016/j.neucom.2023.126719

[19] Pan Y Fu M Cheng B Tao X Guo J. Enhanced deep learning assisted convolutional neural network for heart disease prediction on the internet of medical things platform. *IEEE Access.* (2020) 8:189503–12. 10.1109/ACCESS.2020.3026214

[20] Parvathy V Pothiraj S Sampson J. Automated Internet of Medical Things (IoMT) based healthcare monitoring system. In: Hassanien AE Khamparia A Gupta D Shankar K Slowik A editors. *Cognitive Internet of Medical Things for Smart Healthcare: Services and Applications.* Cham: Springer. (2021). p. 117–28.

[21] Li S Li H Lu Y Zhou M Jiang S Du X Advanced textile-based wearable biosensors for healthcare monitoring. *Biosensors.* (2023) 13:909. 10.3390/bios13100909 37887102 PMC10605256

[22] Wang Q Sun X Liu C Wang C Zhao W Zhu Z Current development of stretchable self-powered technology based on nanomaterials toward wearable biosensors in biomedical applications. *Front Bioeng Biotechnol.* (2023) 11:1164805. 10.3389/fbioe.2023.1164805 37113667 PMC10126507

[23] Tricase A Imbriano A Valentino M Ditaranto N Macchia M Franco C Water-based conductive ink formulations for enzyme-based wearable biosensors. *Adv Sensor Res.* (2024) 3:2300036. 10.1002/adsr.202300036

[24] Nguyen P Soenksen L Donghia N Angenent-Mari N de Puig H Huang A Wearable materials with embedded synthetic biology sensors for biomolecule detection. *Nat Biotechnol.* (2021) 39:1366–74. 10.1038/s41587-021-00950-3 34183860

[25] Yasin A Delaney J Cheng C Pang T. The design and implementation of an IoT sensor-based indoor air quality monitoring system using off-the-shelf devices. *Appl Sci.* (2022) 12:9450. 10.3390/app12199450

[26] Rajagopal S. Lyngdoh DC Songra R Vasan N. IoT-enabled solutions for environmental monitoring in hospitals. *Open Biomed Eng. J.* (2023) 17:e187412072301020. 10.2174/18741207-v17-e230110-2022-HT28-4371-4

[27] Abubeker K Baskar S. Wireless sensor and wireless body area network assisted biosensor network for effective monitoring and prevention of non-ventilator hospital-acquired pneumonia. *Front Sustain Cities.* (2022) 4:1063067. 10.3389/frsc.2022.1063067

[28] Wilson N Calabria C Warren A Finlay A O’Donovan A Passerello G Quantifying hospital environmental ventilation using carbon dioxide monitoring - a Multicentre study. *Anaesthesia*. (2024) 79:147–55. 10.1111/anae.16124 38059394

[29] Alabere I Luckyn BJ Anyanwu FC. Implementation of HCSR04 ultrasonic sensor based internet of things hospital drip bag monitoring and alert system. *J Innov Data Sci Big Data Manage*. (2024) 3:36–50.

[30] Liu X Hu J Zhang H Di B Bian K Song L. Meta-material sensor-based internet of things for environmental monitoring by deep learning: design, deployment, and implementation. *Trans Wireless Comm.* (2023) 22:2462–76. 10.1109/TWC.2022.3211610

[31] Alasmary H. Scalable Digital Health (SDH): an IoT-based scalable framework for remote patient monitoring. *Sensors.* (2024) 24:1346. 10.3390/s24041346 38400504 PMC10893503

[32] Raparla K Pandey N Modh S. Indigenous and disruptive remote patient monitoring devices - a Case Study on AI in healthcare. *SDMIMD J Manag.* (2023) 14:27–34. 10.18311/sdmimd/2023/32513

[33] Ren S McDonald C Corwin D Wiebe D Master C Arbogast K. Response rate patterns in adolescents with concussion using mobile health and remote patient monitoring: observational study. *JMIR Pediatr Parent.* (2024) 7:e53186. 10.2196/53186 38722194 PMC11089889

[34] Kedwan F. Remote patient monitoring system implementation. *J Clin Case Stud Rev Rep.* (2022):1–4. 10.47363/JCCSR/2022(4)232

[35] Vaghasiya J Mayorga-Martinez C Pumera M. Telemedicine platform for health assessment remotely by an integrated nanoarchitectonics FePS3/rGO and Ti3C2-based wearable device. *NPJ Flex Electron.* (2022) 6:73. 10.1038/s41528-022-00208-1 35990769 PMC9376913

[36] Srinivasu P Ijaz M Shafi J Woźniak M Sujatha R. 6G Driven fast computational networking framework for healthcare applications. *IEEE Access.* (2022) 10:94235–48. 10.1109/ACCESS.2022.3203061

[37] Donghua J Zeric N Nkapkop J Nestor T Wang X Awrejcewicz J. A new cross ring neural network: dynamic investigations and application to WBAN. *IEEE Int Things J.* (2023) 10:7143–52. 10.1109/JIOT.2022.3228748

[38] Sodhro A Malokani A Sodhro G Muzammal M Zongwei L. RETRACTED ARTICLE: an adaptive QoS computation for medical data processing in intelligent healthcare applications. *Neural Comput Appl.* (2020) 32:723–34. 10.1007/s00521-018-3931-1

[39] Rehman A Haseeb K Fati S Lloret J Peñalver L. Reliable bidirectional data transfer approach for the internet of secured medical things using ZigBee wireless network. *Appl Sci.* (2021) 11:9947. 10.3390/app11219947

[40] Rehman U Park S Lee S. Secure health fog: a novel framework for personalized recommendations based on adaptive model tuning. *IEEE Access.* (2021) 9:108373–91. 10.1109/ACCESS.2021.3101308

[41] Padma B Babu E. Efficient secure communication in Zigbee network using the DNA sequence encryption technique. *Life.* (2023) 13:1147. 10.3390/life13051147 37240792 PMC10222487

[42] Yu C Ku M Wang L. Joint topology construction and hybrid routing strategy on load balancing for Bluetooth low energy networks. *IEEE Int Things J.* (2021) 8:7101–2. 10.1109/JIOT.2021.3051561

[43] Wang W He D Jia W Chen X Gu T Liu H PRComm: Anti-interference cross-technology communication based on pseudo-random sequence. In: *Proceedings of the 20th International Conference on Information Processing in Sensor Networks.* New York, NY: ACM. (2021). p. 163–75.

[44] Sriram S. Determination of maximum allowable data transfer rate by Bluetooth LET energy method in WSN. In: *Proceedings of the 2023 World Conference on Communication & Computing (WCONF).* 1–6. Raipur: IEEE (2023).

[45] Kozhubaev Y Ovchinnikova E Krotova S Ivanov V Ruide Y. Energy efficient indoor wireless communication techniques based on BLE technology. *E3S Web of Conf.* (2023) 389:7011. 10.1051/e3sconf/202338907011

[46] Verma P Fatima S. Smart healthcare applications and real-time analytics through edge computing. In: Raj P Moy J Abhishek C editors. *Internet of Things Use Cases for the Healthcare Industry.* Cham: Springer International Publishing (2020). p. 241–70. 10.1007/978-3-030-37526-3_11

[47] Ghadi Y Shah A Mazhar T Shahzad T Ouahada K Hamam H. Enhancing patient healthcare with mobile edge computing and 5G: challenges and solutions for secure online health tools. *J Cloud Comput.* (2024) 13:93. 10.1186/s13677-024-00654-4

[48] Hemmati A Raoufi P Rahmani A. Edge artificial intelligence for big data: a systematic review. *Neural Comput Appl.* (2024) 36:11461–94. 10.1007/s00521-024-09723-w

[49] Urblik L Kajati E Papcun P Zolotova I. A modular framework for data processing at the edge: design and implementation. *Sensors.* (2023) 23:7662. 10.3390/s23177662 37688118 PMC10490771

[50] Lakhani Z. The role of cloud integration in healthcare sector: a comprehensive review. *Int J Res Appl Sci Eng Technol.* (2024) 12:4280–5. 10.22214/ijraset.2024.60919

[51] Himanshu Y Punhani A. A perspective to cloud computing in healthcare sector. In: *Proceedings of the 2024 2nd International Conference on Disruptive Technologies (ICDT).* Greater Noida: IEEE (2024). p. 811–7.

[52] Dhote S Baskar S Mohamed Shakeel P Dhote T. *Cloud Computing Assisted Mobile Healthcare Systems using Distributed Data Analytic Model.* Piscataway, NJ: IEEE (2023).

[53] Praveen S Thati B Anuradha C Sindhura S Altaee M Jalil M. A novel approach for enhance fusion based healthcare system in cloud computing. *J Intell Syst Int Things.* (2023) 12:88–100. 10.54216/JISIoT.090106

[54] Zhang H Zhao Y Pang C He J. Splitting large medical data sets based on normal distribution in cloud environment. *IEEE Trans Cloud Comput.* (2020) 8:518–31. 10.1109/TCC.2015.2462361

[55] Prajapati J Prajapati B. Clinical decision support system braced with artificial intelligence: a review. In: Chen J João Manuel R Tavares S editors. *Third International Conference on Image Processing and Capsule Networks.* Cham: Springer (2022). p. 531–40. 10.1007/978-3-031-12413-6_42

[56] Ferdush J Begum M Hossain S. ChatGPT and clinical decision support: scope, application, and limitations. *Ann Biomed Eng.* (2024) 52:1119–24. 10.1007/s10439-023-03329-4 37516680

[57] Demuth S Müller J Quenardelle V Lauer V Gheoca R Trzeciak M Strokecopilot: a literature-based clinical decision support system for acute ischemic stroke treatment. *J Neurol.* (2023) 270:6113–23. 10.1007/s00415-023-11979-6 37668701

[58] Eisenstein M. AI assistance for planning cancer treatment. *Nature.* (2024) 629:S14–6. 10.1038/d41586-024-01431-8 38811703

[59] Savage N. AI’s keen diagnostic eye. *Nature.* (2024) 10.1038/d41586-024-01132-2 Online ahead of print 38637706

[60] Mittermaier M Raza M Kvedar J. Collaborative strategies for deploying AI-based physician decision support systems: challenges and deployment approaches. *NPJ Digit Med.* (2023) 6:137. 10.1038/s41746-023-00889-6 37543707 PMC10404285

[61] Luchini C Pea A Scarpa A. Artificial intelligence in oncology: current applications and future perspectives. *Br J Cancer.* (2022) 126:4–9. 10.1038/s41416-021-01633-1 34837074 PMC8727615

[62] Lenharo M. An AI revolution is brewing in medicine. What will it look like? *Nature.* (2023) 622:686–8. 10.1038/d41586-023-03302-0 37875622

[63] Kiania K Jameii S Rahmani A. Blockchain-based privacy and security preserving in electronic health: a systematic review. *Multimed Tools Appl.* (2023). 10.1007/s11042-023-14488-w Online ahead of print. 36811000 PMC9936121

[64] Wu H Dwivedi A Srivastava G. Security and privacy of patient information in medical systems based on blockchain technology. *ACM Trans Multimedia Comput Commun Appl.* (2021) 17:1–17. 10.1145/3408321

[65] Zarour M Ansari M Alenezi M Sarkar A Faizan M Agrawal A. Evaluating the impact of blockchain models for secure and trustworthy electronic healthcare records. *IEEE Access.* (2020) 8:157959–73. 10.1109/ACCESS.2020.3019829

[66] Zhang C Xu C Sharif K Zhu L. Privacy-preserving contact tracing in 5G-integrated and blockchain-based medical applications. *Comput Stand Interfaces.* (2021) 77:103520. 10.1016/j.csi.2021.103520 33584007 PMC7871810

[67] Zhang D Wang S Zhang Y Zhang Q Zhang Y. A secure and privacy-preserving medical data sharing via consortium blockchain. *Security Commun Netw.* (2022) 2022:1–15. 10.1155/2022/2759787

[68] Alsayegh M Moulahi T Alabdulatif A Lorenz P. Towards secure searchable electronic health records using consortium blockchain. *Network.* (2022) 2:239–56. 10.3390/network2020016

[69] Ayele E Gavriel S Gonzalez J Teeuw W Philimis P Gillani G. Emerging industrial internet of things open-source platforms and applications in diverse sectors. *Telecom.* (2024) 5:369–99. 10.3390/telecom5020019

[70] Rubio J Prieto S. Implementation of a cross-platform development board for embedded internet-of-things systems. *J Telecommun Digital Econ.* (2024) 12:463–85. 10.3316/informit.T2024040300010991472617594

[71] Zhou F Wan X Du X Lu Z Wu J. Design and implementation of an intelligent health management system for nursing homes. In *Proceedings of the 2022 IEEE 7th International Conference on Smart Cloud (SmartCloud).* Shanghai: IEEE (2022). p. 145–50.

[72] Wen M Bai D Lin S Chu C Hsu Y. Implementation and experience of an innovative smart patient care system: a cross-sectional study. *BMC Health Serv Res.* (2022) 22:126. 10.1186/s12913-022-07511-7 35093036 PMC8801128

[73] Boo S Oh H. Perceptions of registered nurses on facilitators and barriers of implementing the AI-IoT-based healthcare pilot project for older adults during the COVID-19 pandemic in South Korea. *Front Public Health.* (2023) 11:1234626. 10.3389/fpubh.2023.1234626 37886046 PMC10598465

[74] Sundas A Badotra S Singh G. Sensor data transforming into real-time healthcare evaluation: a review of internet of things healthcare monitoring applications. In *Proceedings of the 2023 International Conference on Intelligent and Innovative Technologies in Computing, Electrical and Electronics (IITCEE)*, 559–67. Bengaluru: IEEE. (2023).

[75] Liang J Tu J Leung V. Mobile sensor deployment optimization algorithm for maximizing monitoring capacity of large-scale acyclic directed pipeline networks in smart cities. *IEEE Internet things J.* (2020) 8:16083–95.

[76] Jiang J Wang J Wu H Lee C Chou C Wu C A novel sensor placement strategy for an IoT-based power grid monitoring system. *IEEE Int. Things J.* (2020) 7:7773–82. 10.1109/JIOT.2020.2991610

[77] Wang W Zhu Q Gao H. Drift detection of intelligent sensor networks deployment based on graph stream. *IEEE Trans Netw Sci Eng.* (2023) 10:1096–106. 10.1109/TNSE.2022.3227909

[78] Rosa S Kadir E Abbasi Q Almansour A Othman A Siswanto A. Patient monitoring and disease analysis based on IoT wearable sensors and cloud computing. In *Proceedings of the 2022 International Conference on Electrical, Computer, Communications and Mechatronics Engineering (ICECCME).* Maldives: IEEE (2022).

[79] Balakrishnan R Ranganayaki V. Sensor based health monitoring system using embedded technology. In *Proceedings of the 2022 8th International Conference on Advanced Computing and Communication Systems (ICACCS).* Coimbatore: IEEE (2022). p. 1263–7.

[80] Liu Z Cascioli V McCarthy P. Healthcare monitoring using low-cost sensors to supplement and replace human sensation: does it have potential to increase independent living and prevent disease? *Sensors.* (2023) 23:2139. 10.3390/s23042139 36850736 PMC9963454

[81] Harb H Mansour A Nasser A Cruz C Torre Díez I. A sensor-based data analytics for patient monitoring in connected healthcare applications. *IEEE Sensors J.* (2021) 21:974–84. 10.1109/JSEN.2020.2977352

[82] Shafi I Din S Farooq S Díez I Breñosa J Espinosa J Design and development of patient health tracking, monitoring and big data storage using Internet of Things and real time cloud computing. *PLoS One.* (2024) 19:e0298582. 10.1371/journal.pone.0298582 38466691 PMC10927143

[83] Pandey S Singh K Dingankar R Jadhav K Gupta K Yadav R. Prediction of tuberculosis disease progression with AI analysis of clinical data. In: *Proceedings of the 2023 International Conference on Artificial Intelligence for Innovations in Healthcare Industries (ICAIIHI).* Raipur: IEEE (2023). p. 1–6.

[84] Grampurohit S Sagarnal C. Disease prediction using machine learning algorithms. In: *Proceedings of the 2020 International Conference for Emerging Technology (INCET).* Belgaum: IEEE (2020).

[85] Cho S Kim S Kang S Lee K Choi D Kang S Pre-existing and machine learning-based models for cardiovascular risk prediction. *Sci Rep.* (2021) 11:8886. 10.1038/s41598-021-88257-w 33903629 PMC8076166

[86] Glass C Davis R Xiong B Dov D Glass M. The use of Artificial Intelligence (AI) machine learning to determine myocyte damage in cardiac transplant acute cellular rejection. *J Heart Lung Transpl.* (2020) 39:S59. 10.1016/j.healun.2020.01.1250

[87] Yu H. Analysis and prediction of heart disease based on machine learning algorithms. In *Proceedings of the 2023 8th International Conference on Intelligent Computing and Signal Processing (ICSP).* IEEE (2023). Xi’an, p. 1418–23.

[88] Tarumi S Takeuchi W Chalkidis G Rodriguez-Loya S Kuwata J Flynn M Leveraging artificial intelligence to improve chronic disease care: methods and application to pharmacotherapy decision support for type-2 diabetes mellitus. *Methods Inf Med.* (2021) 60(S 01):e32–43. 10.1055/s-0041-1728757 33975376 PMC8294941

[89] Isiaka A Anakwenze V Ilodinso C Anaukwu C Mary-Vin Ezeokoli C Noi S. Harnessing artificial intelligence for early detection and management of infectious disease outbreaks. *Int J Innov Res Dev.* (2024) 13:52–65. 10.24940/ijird/2024/v13/i2/FEB2406

[90] Farrag A Kamel A El-Baraky I. Opportunities and challenges for the application of artificial intelligence paradigms into the management of endemic viral infections: the example of chronic hepatitis C virus. *Rev Med Virol.* (2024) 34:e2514. 10.1002/rmv.2514

[91] Zhuhadar L Lytras M. The application of AutoML techniques in diabetes diagnosis: current approaches, performance, and future directions. *Sustainability.* (2023) 15:13484. 10.3390/su151813484

[92] Kargbo R. Advancements in predictive medicine: NLRP3 inflammasome inhibitors and AI-driven predictive health analytics. *ACS Med Chem Lett.* (2024) 15:331–3. 10.1021/acsmedchemlett.4c00056 38505847 PMC10945530

[93] Alshamrani M. IoT and artificial intelligence implementations for remote healthcare monitoring systems: a survey. *J King Saud Univer Comput Inf Sci.* (2022) 34:4687–701. 10.1016/j.jksuci.2021.06.005

[94] Wang C He T Zhou H Zhang Z Lee C. Artificial intelligence enhanced sensors - enabling technologies to next-generation healthcare and biomedical platform. *Bioelectron Med.* (2023) 9:17. 10.1186/s42234-023-00118-1 37528436 PMC10394931

[95] Martinez-Ortigosa A Martinez-Granados A Gil-Hernández E Rodriguez-Arrastia M Ropero-Padilla C Roman P. Applications of Artificial intelligence in nursing care: a systematic review. *J Nurs Manag.* (2023):3219127. 10.1155/2023/3219127 PMC11919018 40225652

[96] Ni Y Jacob F Achuthan P Wu H Javed F. Leveraging automated search relevance evaluation to improve system deployment: a case study in healthcare. In: *Proceedings of the 31st ACM International Conference on Information & Knowledge Management*, Atlanta, GA: ACM (2022).

[97] Jahanshahi H Kazmi S Cevik M. Auto response generation in online medical chat services. *J Healthc Inform Res.* (2022) 6:344–74. 10.1007/s41666-022-00118-x 35854816 PMC9284963

[98] Bagattini Â Borges J Riera R de Carvalho D. Automation of a tertiary hospital pharmacy drug dispensing system in a lower-middle-income country: a case study and preliminary results. *Explor Res Clin Soc Pharm.* (2022) 6:100151. 10.1016/j.rcsop.2022.100151 35770196 PMC9234249

[99] Odera D. A survey on techniques, methods and security approaches in big data healthcare. *Glob J Eng.* (2023) 14:093–106. 10.30574/gjeta.2023.14.2.0035

[100] Madavarapu J Yalamanchili R Mandhala V. An ensemble data security on cloud healthcare systems. In: *Proceedings of the 2023 4th International Conference on Smart Electronics assnd Communication (ICOSEC).* Trichy: IEEE (2023). p. 680–6.

[101] Basha B. Enhancing healthcare data security using quantum cryptography for efficient and robust encryption. *J Electrical Syst.* (2024) 20:1993–2000. 10.52783/jes.2535

[102] Khan M Khan J Sehito N Mahmood K Ali H Bari I An adaptive enhanced technique for locked target detection and data transmission over internet of healthcare things. *Electronics.* (2022) 11:2726. 10.3390/electronics11172726

[103] Abdelaziz A Alia M. Data security in healthcare systems: integration of information security and information management. *J Cybersecur Inf Manag.* (2023) 11:17–26. 10.54216/JCIM.110202

[104] Naithani P. COMMENTARY: protecting healthcare privacy: analysis of data protection developments in India. *Indian J Med Ethics.* (2023) 9:149–53. 10.20529/IJME.2023.078 38755773

[105] Polubinskaya S. *Big Data in Healthcare: Cybersecsurity and Legal Protection of Personal Data.* Moscow: Russian Academy of Sciences (2023).

[106] McClelland R Harper C. Information privacy in healthcare - The vital role of informed consent. *Eur J Health Law.* (2022) 30:469–80. 10.1163/15718093-bja10097 37582530

[107] Scheibner J Ienca M Vayena E. Health data privacy through homomorphic encryption and distributed ledger computing: an ethical-legal qualitative expert assessment study. *BMC Med Ethics.* (2022) 23:121. 10.1186/s12910-022-00852-2 36451210 PMC9713155

[108] Vedder A Spajiæ D. Moral autonomy of patients and legal barriers to a possible duty of health related data sharing. *Ethics Inf Technol.* (2023) 25: 23. 10.1007/s10676-023-09697-8

[109] Beil-Hildebrand M Smith H. Comparative analysis of advanced practice nursing: contextual and historical influences in North American and German-speaking European countries. *Policy Polit Nurs Pract.* (2022) 23:162–74. 10.1177/15271544221105032 35765227

[110] Wang M Tang J Liu Y Chuang C Shih C. Innovative digital technology adapted in nursing education between Eastern and Western countries: a mini-review. *Front Public Health.* (2023) 11:1167752. 10.3389/fpubh.2023.1167752 37293621 PMC10244494

[111] De Raeve P Davidson P Bergs J Patch M Jack S Castro-Ayala A Advanced practice nursing in Europe-Results from a pan-European survey of 35 countries. *J Adv Nurs.* (2024) 80:377–86. 10.1111/jan.15775 37458267

[112] Efn E. *Advanced Practice Nursing (Apn).* (2023). Available online at: https://efn.eu/?page_id=15936 (accessed July 18, 2024).

[113] Zhang W Ming Y Li G. Research on application of computer big data and mobile communication technology in intelligent nursing service platform. In *Proceedings of the 2022 IEEE International Conference on Electrical Engineering, Big Data and Algorithms (EEBDA).* Piscataway, NJ: IEEE (2022). p. 1–5. 10.1109/EEBDA53927.2022.9744729

[114] Guo C Li H. Application of 5G network combined with AI robots in personalized nursing in China: a literature review. *Front Public Health.* (2022) 10:948303. 10.3389/fpubh.2022.948303 36091551 PMC9449115

[115] Selvaganesan P Russell A Milo T Mahajan A. A seamless healthcare platform for total connectivity throughout the patients medical journey. *Int J Biomed Eng Technol.* (2023) 41:186. 10.1504/IJBET.2023.129196 35009967

[116] Vannieuwenborg F Van der Auwermeulen T Van Ooteghem J Jacobs A Verbrugge S Colle D. Evaluating the economic impact of smart care platforms: qualitative and quantitative results of a case study. *JMIR Med Inform.* (2016) 4:e33. 10.2196/medinform.5012 27799137 PMC5108925

[117] D’Agostino F Almeida M Nomura A Tayaben J. *Nursing informatics within health systems: global comparison.* Milton Park: Taylor and Francis (2022).

[118] van Niekerk L Mathanga D Juban N Castro-Arroyave D Balabanova D. Universities as catalysts of social innovation in health systems in low-and middle-income countries: a multi-country case study. *Infect Dis Poverty.* (2020) 9:90. 10.1186/s40249-020-00684-5 32650822 PMC7353699

[119] Allel K Tapia-Muñoz T Morris W. Country-level factors associated with the early spread of COVID-19 cases at 5, 10 and 15 days since the onset. *Glob Public Health.* (2020) 15:1589–602. 10.1080/17441692.2020.1814835 32894686

[120] von Grünigen S Geissbühler A Bonnabry P. The safe handling of chemotherapy drugs in low- and middle-income countries: an overview of practices. *J Oncol Pharm Pract.* (2021) 28:410–20. 33622088 10.1177/1078155221995539 PMC8832569

[121] Mansour W Boyd A Walshe K. National accreditation programmes for hospitals in the Eastern Mediterranean region: case studies from Egypt, Jordan, and Lebanon. *Int J Health Plann Manag.* (2021) 36:1500–20. 10.1002/hpm.3178 33949699

[122] Dkhimi F Honda A Hanson K Mbau R Onwujekwe O Phuong T Examining multiple funding flows to public healthcare providers in low- and middle-income countries - results from case studies in Burkina Faso, Kenya, Morocco, Nigeria, Tunisia And Vietnam. *Health Policy Plan.* (2022) 38:1139–53. 10.1093/heapol/czad072 37971183 PMC11318792

[123] Abdulrazak B Mostafa Ahmed H Aloulou H Mokhtari M Blanchet F. IoT in medical diagnosis: detecting excretory functional disorders for older adults via bathroom activity change using unobtrusive IoT technology. *Front Public Health.* (2023) 11:1161943. 10.3389/fpubh.2023.1161943 37841702 PMC10574436

[124] Yesmin T Carter M Gladman A. Internet of things in healthcare for patient safety: an empirical study. *BMC Health Serv Res.* (2022) 22:278. 10.1186/s12913-022-07620-3 35232433 PMC8889732

[125] Abril-Jiménez P Merino-Barbancho B Fico G Martín Guirado J Vera-Muñoz C Mallo I Evaluating IoT-based services to support patient empowerment in digital home hospitalization services. *Sensors.* (2023) 23:1744. 10.3390/s23031744 36772784 PMC9919249

[126] Mudawi N. Integration of IoT and fog computing in healthcare based the smart intensive units. *IEEE Access.* (2022) 10:59906–18. 10.1109/ACCESS.2022.3179704

[127] Yongjoh S So-In C Kompunt P Muneesawang P Morien R. Development of an internet-of-healthcare system using blockchain. *IEEE Access.* (2021) 9:113017–31. 10.1109/ACCESS.2021.3103443

[128] Ni Z Jin H Jiang G Wang N Peng A Guo Z A telemedicine-based registration system for the management of renal anemia in patients on maintenance hemodialysis: multicenter study. *J Med Internet Res.* (2019) 21:e13168. 10.2196/13168 31344676 PMC6682288

[129] Wang Z Liu S. Internet of things (IoT) imbedded point-of-care SARS-CoV-2 testing in the pandemic and post-pandemic era. *Biosaf Health.* (2022) 4:365–8. 10.1016/j.bsheal.2022.09.005 36168401 PMC9502434

[130] Shen J Zhang J He Q Pan H Wu Z Nie L Without the need for a second visit&quot; initiative improves patient satisfaction with updated services of outpatient clinics in China. *BMC Health Serv Res.* (2021) 21:267. 10.1186/s12913-021-06260-3 33757490 PMC7986498

[131] Qin A Liu Y Shao C Dong H. Application of intelligent nursing based on cloud computing of internet of things in children with pneumonia and sepsis treated with human gamma globulin. *Eur J Inflamm.* (2023) 21:1721727X231194144. 10.1177/1721727X231194144

[132] Hu M Ling Y Xiong F Xu J. Construction of a three-level enteral nutrition nursing system under the &quot;Internet + medical&quot; mode and an evaluation of its effect in clinical application. *Front Public Health.* (2022) 10:976276. 10.3389/fpubh.2022.976276 36238248 PMC9550871

[133] Jawad MH Bin Hussein Z Zaidan BB Mohamad Jawad FH Mohamad Jawad DH Alredany WHD A systematic literature review of enabling IoT in healthcare: motivations, challenges, and recommendations. *Electronics.* (2022) 11:3223. 10.3390/electronics11193223

[134] Bernardi F Alves D Crepaldi N Yamada D Lima V Rijo R. Data quality in health research: integrative literature review. *J Med Internet Res.* (2023) 25:e41446. 10.2196/41446 37906223 PMC10646672

[135] Syed R Eden R Makasi T Chukwudi I Mamudu A Kamalpousr M Digital health data quality issues: systematic review. *J Med Internet Res.* (2023) 25:e42615. 10.2196/42615 37000497 PMC10131725

[136] Canali S Schiaffonati V Aliverti A. Challenges and recommendations for wearable devices in digital health: data quality, interoperability, health equity, fairness. *PLoS Digit Health.* (2022) 1:e0000104. 10.1371/journal.pdig.0000104 36812619 PMC9931360

[137] Mukherjee S Baral M Chittipaka V Srivastava S. Analysis of internet of things acceptance dimensions in hospitals. In: Mahmood M Raja R Kaur H editors. *Ambient Intelligence and Internet of Things.* Hoboken NJ: John Wiley & Sons, Ltd (2022). p. 189–213. 10.1002/9781119821847.ch6

[138] Madrigal C Mogle J Abbott K Mills W Fick D Scanlon D The association between preference satisfaction and satisfaction with overall care for nursing home residents. *J Aging Soc Policy.* (2022) 34:707–22. 10.1080/08959420.2022.2029265 35491885 PMC9560912

[139] Huo W Yuan X Li X Luo W Xie J Shi B. Increasing acceptance of medical AI: the role of medical staff participation in AI development. *Int J Med Inform.* (2023) 175:105073. 10.1016/j.ijmedinf.2023.105073 37119693 PMC10125218

[140] Abdulkarim Adamu I Umar M Umaru J Dokoro H. Utilizing ICT in sustaining national development using digital economy and IOT. *Int J Wireless Commun Mobile Comput.* (2020) 8:18. 10.11648/j.wcmc.20200802.11

[141] Mathkor D Mathkor N Bassfar Z Bantun F Slama P Ahmad F Multirole of the internet of medical things (IoMT) in biomedical systems for managing smart healthcare systems: an overview of current and future innovative trends. *J Infect Public Health.* (2024) 17:559–72. 10.1016/j.jiph.2024.01.013 38367570

[142] Endres H Indulska M Ghosh A. Unlocking the potential of industrial Internet of Things (IIOT) in the age of the industrial metaverse: business models and challenges. *Industr Mark Manag.* (2024) 119:90–107. 10.1016/j.indmarman.2024.03.006

[143] Guan H. Construction of urban low-carbon development and sustainable evaluation system based on the internet of things. *Heliyon.* (2024) 10:e30533. 10.1016/j.heliyon.2024.e30533 38774092 PMC11106817

[144] Mohammadzadeh N Gholamzadeh M Saeedi S Rezayi S. The application of wearable smart sensors for monitoring the vital signs of patients in epidemics: a systematic literature review. *J Ambient Intell Human Comp*. (2023) 14:6027–41. 10.1007/s12652-020-02656-x 33224305 PMC7664168

[145] Alshamrani M. IoT and artificial intelligence implementations for remote healthcare monitoring systems: a survey. *J King Saud Univ Comp Inform Sci*. (2022) 34:4687–701.

[146] Hu Y Pan Z De Bock M Tan T Wang Y Shi Y A wearable microneedle patch incorporating reversible FRET-based hydrogel sensors for continuous glucose monitoring. *Biosens Bioelectron.* (2024) 262:116542. 10.1016/j.bios.2024.116542 38991372

[147] Mishra S Saha B. Graphene- polymer nanocomposite-based wearable strain sensors for physiological signal monitoring: recent progress and challenges. *Curr Opin Solid State Materials Sci.* (2024) 31:101174. 10.1016/j.cossms.2024.101174

[148] Siao C Chang R Huang H. Robotic arms for telemedicine system using smart sensors and ultrasound robots. *Int Things.* (2024) 27:101243. 10.1016/j.iot.2024.101243

[149] Sikander S Biswas P Kulkarni P. Recent advancements in telemedicine: surgical, diagnostic and consultation devices. *Biomed Eng Adv.* (2023) 6:100096. 10.1016/j.bea.2023.100096

[150] Lu J Ling K Zhong W He H Ruan Z Han W. Construction of a 5G-based, three-dimensional, and efficiently connected emergency medical management system. *Heliyon.* (2023) 9:e13826. 10.1016/j.heliyon.2023.e13826 36895405 PMC9988483

[151] Yeasmin M Al Amin M Joti T Aung Z Azim M. Advances of AI in image-based computer-aided diagnosis: a review. *Array.* (2024) 23:100357. 10.1016/j.array.2024.100357

[152] Zhang J Wu J Qiu Y Song A Li W Li X Intelligent speech technologies for transcription, disease diagnosis, and medical equipment interactive control in smart hospitals: a review. *Comput Biol Med.* (2023) 153:106517. 10.1016/j.compbiomed.2022.106517 36623438 PMC9814440

[153] Muse E Topol E. Transforming the cardiometabolic disease landscape: multimodal AI-powered approaches in prevention and management. *Cell Metab.* (2024) 36:670–83. 10.1016/j.cmet.2024.02.002 38428435 PMC10990799

[154] Tang Z Tang Z Liu Y Tang Z Liao Y. Smart healthcare systems: a new IoT-Fog based disease diagnosis framework for smart healthcare projects. *Ain Shams Eng J.* (2024) 15:102941. 10.1016/j.asej.2024.102941

[155] Li C Wang J Wang Sı Zhang Y. A review of IoT applications in healthcare. *Neurocomputing.* (2024) 565:127017. 10.1016/j.neucom.2023.127017

[156] Fink M Akra B. Comparison of the international regulations for medical devices-USA versus Europe. *Injury.* (2023) 54:110908. 10.1016/j.injury.2023.110908 37365092

[157] Schüller M. Artificial intelligence: new challenges and opportunities for Asian countries. In: Wang L Zhao J editors. *Exchanges and Mutual Learning among Asian Civilizations.* Singapore: Springer Nature. (2023). p. 277–85. 10.1007/978-981-19-7165-5_42

[158] Wan S Chen Y Xiao Y Zhao Q Li L Wu S. Spatial analysis and evaluation of medical resource allocation in China based on geographic big data. *BMC Health Serv Res.* (2021) 21:1084. 10.1186/s12913-021-07119-3 34641850 PMC8508408

[159] Nurse J. The Potential Role of China in Advancing the COVID-19 Policy Framework, Beijng. (2020).

[160] Ge X Zhou R Li Q. 5G NFV-based tactile internet for mission-critical IoT services. *IEEE Internet Things J*. (2020) 7:6150–63. 10.1109/JIOT.2019.2958063

[161] Wijethilaka S Liyanage M Survey on network slicing for internet of things realization in 5G networks. *IEEE Commun Surv Tutor*. (2021) 23:957–994. 10.1109/COMST.2021.3067807

[162] Vaishya R Javaid M Khan IH Haleem A. Artificial intelligence (AI) applications for COVID-19 pandemic. *Diab Metab Syndr Clin Res Rev*. (2020) 14:337–9. 10.1016/j.dsx.2020.04.012 32305024 PMC7195043

[163] Almaiah MA Hajjej F Ali A Pasha MF Almomani O. A novel hybrid trustworthy decentralized authentication and data preservation model for digital healthcare IoT based CPS. *Sensors* (2022) 22:1448. 10.3390/s22041448 35214350 PMC8875865

[164] Taimoor N Rehman S. Reliable and resilient AI and IoT-based personalised healthcare services: a survey. *IEEE Access*. (2022) 10:535–63. 10.1109/ACCESS.2021.3137364

[165] Panagiotidis P. Blockchain in education-the case of language learning. *Eur J Educ*. (2022) 5:66–82. 10.26417/443gjm83

[166] Simpson K Nham W Thariath J Schafer H Greenwood-Eriksen M Fetters MD How health systems facilitate patient-centered care and care coordination: a case series analysis to identify best practices. *BMC Health Serv Res*. (2022) 22:1448. 10.1186/s12913-022-08623-w 36447273 PMC9710067

[167] Al-Rawashdeh M Keikhosrokiani P Belaton B Alawida M Zwiri A. Effective factors for the adoption of IoT applications in nursing care: a theoretical framework for smart healthcare. *J Build Eng.* (2024) 89:109012. 10.1016/j.jobe.2024.109012

[168] Yelne S Chaudhary M Dod K Sayyad A Sharma R. Harnessing the power of AI: a comprehensive review of its impact and challenges in nursing science and healthcare. *Cureus.* (2023) 15:e49252. 10.7759/cureus.49252 38143615 PMC10744168

[169] Maina A Singh U. *Exploring the Scope of Policy Issues Influencing IoT Health and Big Data: A Structured Review.* Florida: Apple Academic Press (2022).

[170] Casarosa F. Cyber security of Internet of Things in the health sector: understanding the applicable legal framework. *Comput Law Sec Rev.* (2024) 53:105982. 10.1016/j.clsr.2024.105982

[171] Rathee G Sharma A Saini H Kumar R Iqbal R. A hybrid framework for multimedia data processing in IoT-healthcare using blockchain technology. *Mult Tools Appl.* (2020) 79:9711–33. 10.1007/s11042-019-07835-3

[172] Sadhu P Yanambaka V Abdelgawad A Yelamarthi K. Prospect of internet of medical things: a review on security requirements and solutions. *Sensors.* (2022) 22:5517. 10.3390/s22155517 35898021 PMC9371024

[173] Ghosh A Saha R Misra S. Persistent service provisioning framework for IoMT based emergency mobile healthcare units. *IEEE J Biomed Health Inform.* (2022) 26:5851–8. 10.1109/JBHI.2022.3172624 35511843

[174] Fink M Akra B. Comparison of the international regulations for medical devices-USA versus Europe. *Injury*. (2023) 54:110908. 10.1016/j.injury.2023.110908 37365092

[175] Raul AC (ed.). *The Privacy, Data Protection and Cybersecurity Law Review*. Law Business Research Limited (2021).

[176] Szarfman A Levine JG Tonning JM Weichold F Bloom JC Soreth JM Recommendations for achieving interoperable and shareable medical data in the USA. *Commun Med*. (2022) 2:86. 10.1038/s43856-022-00148-x 35865358 PMC9293957

